# Critical Overview of Molecular Insights into Osteoarthritis and Therapeutic Targets: Cytokines, RANKL, MMPs, Adipokines and Phosphate Dysregulation

**DOI:** 10.3390/ijms27125292

**Published:** 2026-06-11

**Authors:** Mikołaj Bugajewski, Artur Stolarczyk, Maja Matysek, Jakub Piotr Adamus, Aleksandra Poszytek, Leszek Pączek

**Affiliations:** 1 Clinical Immunology Student Scientific Association, Medical University of Warsaw, Nowogrodzka 59, 02-006 Warsaw, Poland; mikolajbugajewski@gmail.com (M.B.); majka.mtsk@gmail.com (M.M.); jadamus.md@gmail.com (J.P.A.); aposzytek22@gmail.com (A.P.); 2 Department of Orthopedics and Rehabilitation, Medical University of Warsaw, 02-091 Warsaw, Poland; 3 Department of Clinical Immunology, Medical University of Warsaw, 02-006 Warsaw, Poland; leszek.paczek@wum.edu.pl; 4 Institute of Biochemistry and Biophysics, Polish Academy of Sciences, ul. Pawińskiego 5A, 02-106 Warsaw, Poland

**Keywords:** osteoarthritis, cytokines, adipokines, RANK/RANKL/OPG, matrix metalloproteinases, ADAMTS, subchondral bone remodeling, obesity, basic calcium phosphate crystals, pyrophosphate (PPi)

## Abstract

Osteoarthritis (OA) is a highly prevalent joint disorder traditionally considered a consequence of mechanical cartilage wear; however, it is now recognized as a complex, multifactorial disease driven by interconnected molecular and cellular mechanisms. This narrative review synthesizes current knowledge on key pathogenic pathways underlying OA progression, with a focus on inflammatory signaling, subchondral bone remodeling, and dysregulation of mineral metabolism. Chronic low-grade inflammation promotes catabolic responses in chondrocytes and contributes to cartilage degradation. In addition, obesity influences OA pathogenesis through both biomechanical loading and adipokine-mediated inflammatory mechanisms. Alterations in the receptor activator of nuclear factor kappa-B/receptor activator of nuclear factor kappa-B ligand/osteoprotegerin (RANK/RANKL/OPG) axis disrupt bone homeostasis and promote pathological subchondral remodeling, while imbalances in inorganic phosphate metabolism contribute to crystal deposition and further joint damage. These processes interact synergistically, driving disease progression. Current therapeutic strategies remain largely symptomatic and do not adequately target underlying molecular drivers. A deeper understanding of these mechanisms may facilitate the development of disease-modifying therapies.

## 1. Introduction

Osteoarthritis (OA) is a common degenerative joint disease characterized by the progressive breakdown of articular cartilage and reactive changes in the subchondral bone. It is also associated with joint space narrowing, osteophyte formation, subchondral bone sclerosis, and chronic inflammation. These changes lead to persistent pain, reduced mobility, and are a major cause of disability, particularly in older adults [1].

In 2020, approximately 595 million people worldwide were living with OA. This represented 7.6% of the global population. Compared with 1990, the number of cases was 132.2% higher. Knee OA is the most common site of disease. It contributed the most to the overall OA burden, and its age-standardized prevalence rate increased between 1990 and 2019. During the same period, the age-standardized prevalence rate of hip OA also increased [2]. For hip OA diagnosed using the radiographic Kellgren–Lawrence grade ≥ 2 criterion, the pooled worldwide prevalence was estimated at 8.55% (95% CI, 4.85–13.18) [3].

Knee OA and hip OA should not be treated as fully interchangeable conditions. They share several pathogenic mechanisms. However, they differ in prevalence, prognosis, biomechanics, clinical presentation, pain characteristics, and some aspects of molecular pathophysiology. Knee OA is more prevalent. It is also more often associated with female sex, malalignment, joint instability, and higher knee joint loading. In contrast, hip OA is less prevalent and shows a more similar distribution between men and women. Hip OA is also more often associated with restricted range of motion, different pain characteristics, and a shorter time to joint replacement. Molecular differences have also been reported. These include distinct cytokine profiles and differences in synovial inflammatory cell composition between hip and knee OA. Therefore, findings from knee OA should not be automatically extrapolated to hip OA. In this review, site-specific evidence is distinguished where available [4].

Recent OA research increasingly emphasizes disease heterogeneity and patient stratification. Molecular endotypes may define OA subgroups more precisely than clinical phenotypes alone. Biochemical markers of cartilage turnover, bone remodeling, and other tissue-specific processes may help identify treatable OA endotypes and improve patient selection for disease-modifying clinical trials [5]. Recent studies also point to additional molecular factors involved in joint homeostasis, including trace elements, which may affect inflammation, redox balance, extracellular matrix synthesis, and the function of chondrocytes, osteoblasts, osteoclasts, and synoviocytes [6]. Advances in cartilage biomechanics and diagnostic methods may further improve early detection and monitoring of OA progression. Conventional imaging remains important, but emerging methods can provide additional information about the biomechanical and biochemical status of articular cartilage [7]. Together, these developments support the view of OA as a heterogeneous whole-joint disease driven by interconnected molecular, mechanical, inflammatory, and metabolic pathways.

Far from a simple wear-and-tear condition, OA is now recognized as a whole-joint disease in which cartilage degeneration is accompanied by pathological changes in subchondral bone and the synovium. Chronic low-grade inflammatory processes and metabolic alterations further drive the progression of joint degeneration in OA [3,8].

In OA, the subchondral bone undergoes abnormal turnover and remodeling, driven in part by dysregulation of the receptor activator of nuclear factor kappa-B/receptor activator of nuclear factor kappa-B ligand/osteoprotegerin (RANK/RANKL/OPG) pathway and the action of pro-inflammatory cytokines. At the same time, articular cartilage degradation is mediated by proteolytic enzymes, including matrix metalloproteinases (MMPs) and A Disintegrin and Metalloproteinase with Thrombospondin Motifs (ADAMTS), within an inflammatory milieu [9,10].

Osteoarthritis is influenced not only by local joint processes but also by systemic factors. Chronic low-grade inflammation and metabolic disturbances associated with obesity contribute to disease onset and progression [11].

Early diagnosis of OA remains challenging, as clinical symptoms often appear only after substantial joint damage has already occurred [12]. Therefore, there is an urgent need for sensitive biomarkers that can enable the earlier detection of the disease and better monitoring of its progression.

The aim of this narrative review is to critically synthesize current evidence on the molecular mechanisms driving OA progression. We also assess their potential as therapeutic targets. We examine inflammatory cytokine signaling and extracellular matrix degradation mediated by MMPs and ADAMTS. We then discuss subchondral bone remodeling involving the RANK/RANKL/OPG axis. In addition, we address obesity-associated adipokine signaling and inorganic pyrophosphate–inorganic phosphate (PPi–Pi) imbalance with basic calcium phosphate (BCP) crystal deposition. Finally, we discuss current and emerging therapeutic strategies targeting these interconnected mechanisms.

## 2. Materials and Methods

This narrative review summarizes current evidence on the molecular mechanisms underlying osteoarthritis, with a focus on inflammatory cytokine signaling, extracellular matrix degradation, subchondral bone remodeling, adipokine signaling, and phosphate metabolism.

A literature search was conducted using PubMed, Scopus, and Web of Science. The search focused primarily on articles published between 2000 and 2025. Earlier landmark studies and highly relevant recent publications were also included when they provided essential mechanistic, methodological, or translational context. The search strategy included combinations of the following keywords: “osteoarthritis,” “cytokines,” “IL-1β,” “TNF-α,” “RANKL,” “OPG,” “matrix metalloproteinases,” “MMP,” “ADAMTS,” “aggrecanases,” “adipokines,” “subchondral bone remodeling,” “basic calcium phosphate,” “BCP crystals,” “pyrophosphate,” “phosphate metabolism,” “biomarkers,” “molecular endotypes,” and “disease-modifying osteoarthritis drugs.”

Original research articles, experimental studies, observational clinical studies, clinical trials, and relevant review articles published in English were considered. Studies were included if they addressed molecular or cellular mechanisms relevant to OA pathogenesis, biomarkers of OA progression, or therapeutic targets related to the pathways discussed in this review. Both preclinical studies, including in vitro and animal models, and clinical studies were included to provide a translational perspective.

Publications were excluded if they were not related to OA, focused exclusively on other arthritides without relevance to OA, lacked mechanistic or therapeutic relevance to the review topic, were not available in English, or were conference abstracts, editorials, letters, or non-peer-reviewed materials. Studies focusing on knee OA were included when relevant to general OA pathophysiology. Where available, evidence specific to knee OA and hip OA was distinguished.

The database searches identified approximately 4000 potentially relevant records across PubMed, Scopus, and Web of Science. After preliminary screening of titles and abstracts, approximately 300 full-text articles were considered for detailed evaluation. Ultimately, 138 publications were included in the reference list based on their relevance to the molecular pathways and therapeutic targets discussed in this narrative review. The selected literature was qualitatively analyzed and synthesized to describe key pathogenic mechanisms, assess the consistency and translational relevance of available evidence, and discuss potential therapeutic implications. No formal meta-analysis or risk-of-bias assessment was performed, as the aim of this work was to provide an integrative, mechanism-based narrative overview rather than a systematic review.

## 3. Pathogenesis of Osteoarthritis

OA was once considered primarily a non-inflammatory degenerative condition caused by mechanical wear. However, it is now well established that the development of OA is driven mainly by active biological processes, particularly inflammation and dynamic tissue remodeling [13,14]. The underlying causes of inflammation and remodeling in the joint are the combined effect of mechanical stress, abnormal joint mechanics, and risk factors. These contributors together induce pro-inflammatory mediators and proteases driving joint destruction [15]. The pathogenesis involves a complex interplay between joint tissues, including articular cartilage, subchondral bone, and the synovial membrane. At the cartilage–bone interface, subchondral bone remodeling is closely associated with progressive cartilage degeneration. Increased subchondral bone thickness is linked to more severe cartilage damage [16].

### 3.1. Changes in Joint Tissues in Osteoarthritis—Articular Cartilage, Subchondral Bone, Synovial Membrane

The first degenerative changes are focal fibrillations on the articular cartilage surface. They arise from abnormal joint loading caused by dysplasia or injury, which in turn triggers an aberrant repair response. This disturbed repair process is accompanied by the activation of inflammatory signaling pathways within the joint microenvironment [17,18]. The inflammatory mediators (mainly nuclear factor kappa-light-chain-enhancer of activated B cells (NF-κB) and possibly hypoxia-inducible factor 2 alpha (HIF-2α) and metal regulatory transcription factor 1 (MTF1)), together with mechanical and oxidative stress, alter the functional profile of cell populations within the joint [19,20,21].

Upon stimulation, cell populations within the joint, including chondrocytes, synoviocytes, and mononuclear cells, begin to produce proteolytic enzymes and cytokines, including interleukin-6 (IL-6), interleukin-8 (IL-8), interleukin-10 (IL-10), interleukin-1β (IL-1β), and tumor necrosis factor alpha (TNF-α). Chondrocytes also undergo activation and hypertrophic differentiation [22,23]. Among these cytokines, IL-1β and TNF-α promote the synthesis of matrix metalloproteinases (MMPs), particularly MMP-1 and MMP-3 [24].

As OA progresses, extensive degradation of the cartilage extracellular matrix becomes evident. This process is driven mainly by pro-inflammatory cytokines, including IL-1β and tumor necrosis factor (TNF-α). These cytokines stimulate the production of matrix-degrading enzymes [25]. Since chondrocytes maintain the cartilage matrix, ECM loss is closely linked to chondrocyte dysfunction. Activated chondrocytes shift toward a catabolic phenotype. They produce inflammatory mediators and proteolytic enzymes. They also become more prone to apoptosis. As a result, areas with reduced cellularity appear within the cartilage. This is followed by progressive cartilage loss. Cartilage loss reduces the joint space and increases mechanical stress within the joint [26].

Progressive cartilage degeneration in OA is closely associated with anabolic remodeling of the subchondral bone. Further development of OA is characterized by increased osteoblast activity, thickening of the subchondral bone plate, and increased trabecular bone volume [27,28]. Studies show that in patients with hip OA, subchondral bone remodeling was markedly increased compared to controls, as evidenced by a significantly higher number of osteoblasts per bone perimeter (16.4 ± 10.2 mm^−1^ vs. 3.7 ± 4.5 mm^−1^, *p* < 0.001) and a substantial increase in osteoid surface [29]. In parallel, bone cysts and cartilage outgrowths form and eventually ossify into osteophytes [15].

**Figure 1:**
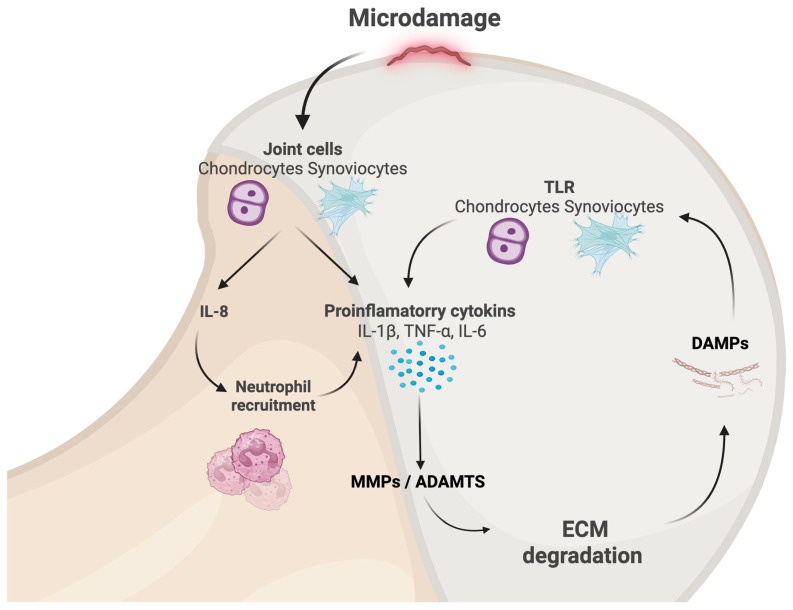
Vicious cycle of inflammation and extracellular matrix degradation in osteoarthritis. Mechanical microdamage in subchondral bone and cartilage initiates the release of damage-associated molecular patterns (DAMPs), which activate Toll-like receptor (TLR) signaling in chondrocytes and synoviocytes. This leads to increased production of pro-inflammatory cytokines, including interleukin-1β (IL-1β), tumor necrosis factor alpha (TNF-α), and interleukin-6 (IL-6), as well as chemokines such as interleukin-8 (IL-8), promoting neutrophil recruitment. These mediators upregulate matrix-degrading enzymes, including matrix metalloproteinases (MMPs) and A Disintegrin and Metalloproteinase with Thrombospondin Motifs (ADAMTSs), resulting in extracellular matrix (ECM) degradation. Degradation products further amplify DAMP signaling, sustaining a self-perpetuating inflammatory loop that drives osteoarthritis progression. Created in BioRender. Bugajewski, M. (2026) https://BioRender.com/c9pnusb.

In OA, the synovium is characterized by low-grade inflammation and infiltration of leukocytes within the subintimal layer. These cells include macrophages, T lymphocytes, B lymphocytes, and neutrophils. Synovitis has been associated with radiographic progression, cartilage damage, and pain in OA [30]. Leukocytes, particularly synovial macrophages, together with activated synovial fibroblasts, secrete cytokines such as IL-6, IL-1β, and TNF-α, which amplify the inflammatory process in OA [30,31]. Synovial inflammation is therefore not only a secondary response to cartilage damage. It also actively contributes to OA progression. Activated synovial cells can release chemokines, prostaglandins, and matrix-degrading enzymes. These mediators further promote cartilage catabolism. This chronic low-grade inflammation establishes a vicious cycle (Figure 1). Pro-inflammatory cytokines stimulate chondrocytes to catabolize the cartilage matrix. This leads to the release of breakdown products such as fibronectin fragments, hyaluronan, and collagen peptides. These products act as damage-associated molecular patterns (DAMPs). They engage pattern-recognition receptors, including Toll-like receptors (TLR), on synovial and cartilage cells. This, in turn, perpetuates the inflammatory response and contributes to OA progression [30,32,33].

### 3.2. Subchondral Bone Remodeling and the RANK/RANKL/OPG Pathway

Osteoarthritis of weight-bearing joints involves not only cartilage degeneration but also active remodeling of the subchondral bone, which plays a central role in the development of hip OA [29]. In animal models, repetitive end-loading of bone induces microdamage that triggers apoptosis in nearby osteocytes [34]. Importantly, apoptosis occurs prior to osteoclastic resorption and co-localizes with it [35]. Microdamage elicits a distance-dependent cellular response. Osteocytes at the lesion border activate caspase-3 and undergo apoptosis. By contrast, cells located further away upregulate RANKL expression [36]. Taken together, these observations highlight osteocytes as pivotal regulators that locally orchestrate the onset of bone remodeling [37].

Both inside OA joint and in surrounding tissues, RANKL is produced by multiple cell types including osteoblast, stromal lineage cells, articular chondrocytes, synovial fibroblasts, and activated T-cells. Among them, osteocytes are the predominant RANKL source in mature bone [31,37,38,39].

**Figure 2:**
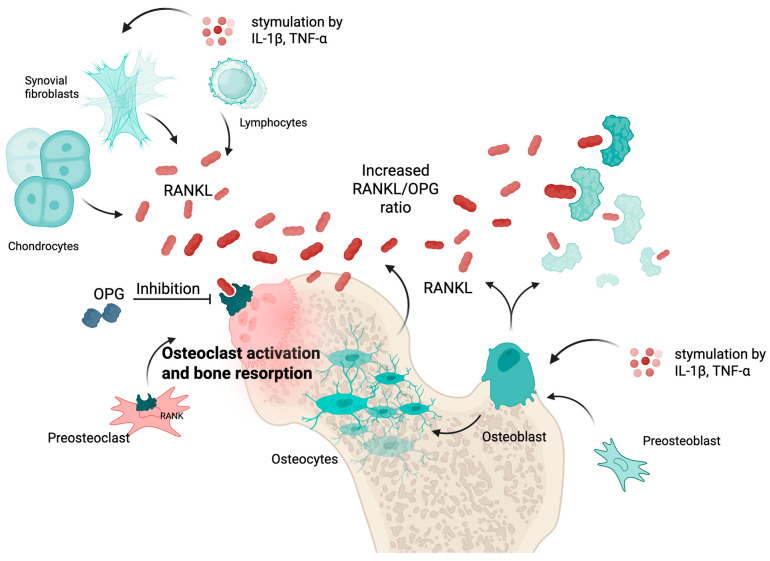
The RANK/RANKL/OPG axis in osteoarthritis. RANKL binds to RANK on osteoclast precursors, promoting osteoclast differentiation and bone resorption. OPG acts as a decoy receptor that inhibits RANKL–RANK interaction. Dysregulation of this axis contributes to subchondral bone remodeling in osteoarthritis. Created in BioRender. Bugajewski, M. (2026) https://BioRender.com/8oowmw1.

The RANK/RANKL/OPG signaling pathway constitutes a central mediator of bone remodeling, as illustrated in Figure 2. Binding of RANKL to its receptor RANK on osteoclast precursors promotes osteoclast differentiation and bone resorption. In contrast, OPG acts as a decoy receptor that inhibits the RANK-RANKL interaction and thereby limits bone resorption [10]. Evidence from human studies shows that osteoarthritic subchondral bone exhibits an imbalance in RANKL and OPG expression. In subsets of osteoblasts with elevated turnover, those derived from OA subchondral bone secrete relatively higher amounts of RANKL and lower amounts of OPG compared with osteoblasts from non-arthritic bone [40]. Similarly, gene expression profiling of subchondral bone from OA knees found significantly elevated RANKL expression (approximately 1.63-fold; *p* = 0.048) with relatively unchanged OPG, resulting in decreased OPG/RANKL ratio compared with normal bone [41]. Analogically, since RANK/RANKL/OPG-mediated remodeling is common to all load-bearing joints, and hip OA features both osteoblast dysfunction and high-turnover subchondral lesions, a similarly reduced OPG/RANKL ratio is plausible.

In male patients with primary hip OA undergoing total hip replacement, trabecular RANKL mRNA expression in the trochanteric region showed strong positive correlations with histomorphometric markers of bone turnover. Notably, these associations were observed for osteoclast number (r = 0.72, *p* = 0.002) and osteoid surface (r = 0.66, *p* = 0.006). Additionally, RANKL mRNA levels were inversely correlated with trabecular bone volume (bone volume/tissue volume (BV/TV); r = −0.70, *p* = 0.007). Circulating RANKL was also negatively correlated with bone RANKL mRNA (r = −0.70; *p* = 0.007), underscoring the pivotal role of the RANKL/OPG system in subchondral bone remodeling [42]. Accordingly, joint measurement of bone RANKL/OPG expression and serum RANKL (±OPG) could constitute a biomarker panel for indexing subchondral bone remodeling in hip OA.

Apart from mechanical factors, another potent driver of RANKL-dependent subchondral bone remodeling in hip OA is the local surge of pro-inflammatory mediators [43]. In vitro studies show that exposure of human articular chondrocytes to catabolic cytokines such as TNF-α or IL-1β significantly increases the production of membrane-bound RANKL (as well as OPG) by these cells [44]. Interestingly, IL-1β stimulation leads to the appearance of RANK on a fraction of osteoarthritic chondrocytes [44], potentially making them a direct target for RANKL. Similar cytokine-driven upregulation of RANKL has been reported in synovial fibroblasts, further expanding the spectrum of joint-resident cells contributing to bone resorption [45]. Taken together, these findings indicate that an inflammatory milieu amplifies osteoclastogenic signaling and accelerates subchondral bone remodeling in OA.

Collectively, both clinical and experimental observations converge on the idea that RANKL is not merely associated with, but causally drives OA-related subchondral bone changes. Chondrocyte-derived RANKL has been shown to drive osteoclast formation; for instance, articular chondrocytes isolated from OA cartilage can induce robust osteoclast differentiation when co-cultured with monocyte precursors, an effect that is further enhanced when RANKL secretion is stimulated by prostaglandin E_2_ (PGE_2_) [38]. Consistent findings were obtained with osteoblasts: those derived from OA subchondral bone, characterized by a reduced OPG-to-RANKL ratio, were more potent in promoting osteoclast differentiation in vitro than osteoblasts from non-arthritic bone [40].

These findings are also supported by in vivo studies. One study in antigen-induced arthritic rabbits demonstrated that animals with higher RANKL expression in their cartilage exhibited greater subchondral bone loss. RANKL levels showed a strong inverse correlation with subchondral bone mineral density (r = −0.891, *p* < 0.001), and the RANKL/OPG ratio was also negatively correlated with bone mineral density (r = −0.736, *p* = 0.01) [38]. Taken together, these results suggest that increased RANKL activity in OA promotes osteoclastogenesis. This process likely contributes to the altered bone remodeling observed in OA joints.

Current evidence supports the involvement of the RANK/RANKL/OPG axis in subchondral bone remodeling in OA. Its therapeutic relevance remains mainly mechanistic or preclinical. Differences between knee OA and hip OA should be considered when interpreting these findings.

### 3.3. Cytokines in Osteoarthritis

In OA, pro-inflammatory cytokines play a central role in disease progression. Among these, IL-1β and TNF-α are regarded as key mediators. IL-1β is primarily associated with cartilage destruction, while TNF-α drives the broader inflammatory cascade [25].

In addition to IL-1β and TNF, a variety of other cytokines, such as IL-6, IL-15, IL-17, IL-18, IL-21, leukemia inhibitory factor (LIF) and the chemokine IL-8, have been associated with the development of OA and could be potential therapeutic targets. They stimulate the release of several catabolic and inflammatory mediators that contribute to joint degradation [25,46].

Conversely, anti-inflammatory cytokines such as IL-4, IL-10 and IL-13 are present but appear to be insufficient to counteract the dominant pro-inflammatory environment [47]. Indeed, OA chondrocytes exhibit reduced expression of the regulatory cytokines IL-4, IL-10, and transforming growth factor beta (TGF-β) activity compared to increased IL-1 and TNF-α expression [48].

TGF-β has a wide range of effects. It can promote joint degradation, while also exerting anti-inflammatory and protective functions. It signals through multiple pathways, including SMAD family members 2 and 3 (SMAD2/3), SMAD family members 1, 5, and 8 (SMAD1/5/8), mitogen-activated protein kinase (MAPK), and phosphoinositide 3-kinase/protein kinase B (PI3K/AKT). These pathways collectively regulate cartilage homeostasis, joint development, and OA progression [49,50,51].

Overall, the cytokine profile in OA is strongly skewed toward a catabolic and inflammatory phenotype.

#### 3.3.1. Pro-Inflammatory Cytokines in Osteoarthritis

TNF-α and IL-1β generally act in a similar way and often work together in OA, activating the same group of intracellular signaling pathways and enhancing inflammation and tissue breakdown in the joints [47]. Furthermore, TNF-α and IL-1β have been shown to affect mitochondrial function in human chondrocytes. They decreased mitochondrial complex I activity, reduced ATP production, and induced mitochondrial depolarization. They also increased the expression of Bcl-2 family proteins. Inhibition of complex I by rotenone increased Bcl-2 expression and reduced proteoglycan content [52].

In a study by Benito et al., synovial fluid from patients with knee pain, normal radiographs and arthroscopic evidence of early OA was compared with that from patients with advanced OA undergoing knee arthroplasty (late OA). The researchers found that patients with early OA had higher levels of IL-1β and TNF-α in their synovial fluid than patients with late OA [53].

These cytokines are mostly produced by activated chondrocytes, mononuclear cells and synoviocytes [54], and their levels are elevated in the synovial fluid, synovial membrane, peripheral blood and cartilage of OA patients [14]. They stimulate the release of several catabolic and inflammatory mediators that contribute to joint degradation [25]. The destruction of intraarticular structures is also enhanced by reactive oxygen species (ROS), which are thought to be increased by the aforementioned cytokines [55].

In addition to IL-1β and TNF-α, a number of other cytokines contribute to the pathophysiology of OA by promoting cartilage degradation, synovial inflammation and subchondral bone remodeling. IL-1β and TNF-α have been reported to stimulate IL-8 production in joint tissues [56,57]. Being a very potent chemokine, IL-8 facilitates neutrophil recruitment and may indirectly accelerate cartilage damage by increasing local inflammation [14]. Studies have reported higher levels of IL-8 in late-stage hip OA compared to earlier stages of the disease [58,59].

IL-15 and IL-18 are elevated in OA joints compared with healthy controls, and increased plasma IL-8 together with higher synovial fluid IL-18 levels may contribute to OA pathogenesis by promoting MMP-3 activation [60]. Additionally, in a study regarding human cholesterol-loaded macrophages, researchers found that IL-8 inhibited the expression of tissue inhibitor of metalloproteinase-1 (TIMP-1) by up to 30% relative to the control [61]. This result was discussed in the context of atherosclerosis; nevertheless, it might have a tangible impact on OA dynamics, as the imbalance between MMPs and TIMPs leads to cartilage degradation and arthropathies [62].

IL-18 in particular increases with disease severity and promotes matrix degradation by inducing MMPs [23]. There is also evidence of a contribution of IL-17 to OA progression. This cytokine was detectable in the synovial fluid in approximately 9% of OA patients and was linked to a specific biological profile of intraarticular changes [63]. A meta-analysis based on observational studies also addressed IL-17 in the OA population and found that its gene polymorphism was associated with an increased risk of disease development [64]. IL-17 induces cartilage breakdown and ADAMTS-mediated degradation of aggrecan, a key structural component of cartilage [65]. Having been recognized as an important factor in pathogenesis of multiple joint disease, such as rheumatoid arthritis and ankylosing spondylitis, this mediator should also be scrutinized in the future of OA research [66,67].

Another molecule able to influence neutrophils is granulocyte-macrophage colony-stimulating factor (GM-CSF), which was shown to promote endothelial adhesion of these cells [68] and to stimulate their viability and pro-inflammatory functions [69,70]. Acting on bone marrow and inducing progenitor cells, GM-CSF promotes their proliferation and terminal differentiation into neutrophils, monocytes, and ultimately macrophages. This is particularly important, as activated macrophages were present and detected by Single-Photon Emission Computed Tomography/Computed Tomography (SPECT/CT) in over 75% of joints in patients suffering from OA [71].

The findings show that removing macrophages from synovial cell cultures significantly reduces the levels of IL-1, TNF-α, MMPs and aggrecanases. These factors are all known to contribute to joint destruction in OA [72].

Activated macrophages are also a well-documented source of IL-6, a pleiotropic cytokine which can negatively affect cartilage tissue [73]. The level of IL-6 in the synovial fluid of patients with OA is elevated [74].

Low innate production of the pro-inflammatory cytokines IL-1β and IL-6 appears to offer protection against OA. Participants with the lowest IL-1β levels were over 11 times more likely to be OA-free, while those with low IL-6 were nearly seven times more likely. Low IL-6 and IL-1 receptor antagonist (IL-1RA) production were linked to an absence of hand OA, while low IL-1β was associated with an absence of knee OA. Overall, these results emphasize the pivotal role of IL-1β and IL-6-driven inflammation in OA development [75].

Experimental studies in mice suggest a more complex role for IL-6. Male mice with an IL-6 knockout gene developed more severe spontaneous OA than wild-type controls during aging. This phenotype was characterized by reduced proteoglycan synthesis and a greater decrease in bone mineral density in the total knee joint. These findings suggest that IL-6 may have a protective role in age-related OA in male mice [76]. These findings suggest that the role of IL-6 in OA may depend on the context and the species. Interestingly, Li et al. reported that IL-1β, IL-6, and TNF-α are major contributors to pain in the early stages of knee osteoarthrosis [77].

#### 3.3.2. Anti-Inflammatory Cytokines in Osteoarthritis

The primary anti-inflammatory cytokines involved in the pathogenesis of OA are IL-4, IL-10 and IL-13 [47]. IL-4 and IL-13 bind to the IL-4 receptor alpha chain (IL-4Rα). The fact that they share this receptor underlies their overlapping biological functions [78]. Signaling through the IL-4 receptor alpha (IL-4Rα), Janus kinase 1 (JAK1), and signal transducer and activator of transcription 3 and 6 (STAT3/STAT6) pathway regulates the expression of multiple genes involved in inflammatory responses. Notably, serum levels of soluble IL-4Rα are elevated in patients with OA compared with healthy controls. By binding IL-4, soluble IL-4Rα may reduce its availability for membrane-bound IL-4 receptors and thereby limit its protective effects on chondrocytes [79].

IL-4 exerts pronounced chondroprotective effects, including inhibiting proteoglycan degradation in articular cartilage by suppressing MMP secretion [80]. Furthermore, IL-4 alone or in combination with IL-10 has been shown to prevent blood-induced cartilage damage [81]. In addition to modulating cytokine production, IL-4 can increase the expression of TNFα receptors (TNF-R1 and TNF-R2) and reduce the secretion of other inflammatory mediators, such as PGE_2_, cyclooxygenase-2 (COX-2), phospholipase A_2_ (PLA_2_) and inducible nitric oxide synthase (iNOS) [47,82].

Elevated levels of IL-13 have been detected in the synovial fluid of OA patients [83]. IL-13 can increase the secretion of periostin by OA synovial fibroblasts via STAT6 activation [84]. Increased periostin levels may boost the expression of MMP-2 and MMP-3 in OA synovial fibroblasts, which could affect extracellular matrix remodeling in the joint [85]. In a model of immune complex-induced arthritis, local overexpression of IL-13 was found to significantly reduce both chondrocyte death and the MMP-dependent degradation of aggrecan in cartilage, despite the fact that joint inflammation was increased [86].

IL-10, which is structurally related to interferons [87], is a key anti-inflammatory cytokine that plays a role in the pathogenesis of OA. Chondrocytes express both IL-10 and its receptor complex (IL-10R1/IL-10R2), enabling autocrine and paracrine signaling. Upon binding to its receptors, IL-10 activates the JAK1/TYK2-STAT3 pathway, which suppresses the expression of pro-inflammatory genes and induces the production of anti-inflammatory mediators such as the IL-1RA and the tissue inhibitor of metalloproteinases-1 (TIMP-1) [47,88].

IL-10 also promotes anabolic cartilage responses by stimulating the synthesis of type II collagen and aggrecan, while protecting chondrocytes from apoptosis [89]. It also activates the SMAD1/5/8 and extracellular signal-regulated kinase 1 and 2 (ERK1/2) pathways, enhancing the expression of bone morphogenetic proteins (BMPs) 2 and 6, which are essential for cartilage regeneration [90]. IL-10 also reduces the inflammatory effects of TNF-α in OA synovial fibroblasts, including reducing PGE_2_ production and decreasing COX-2 expression [82].

Cytokines are important mediators of OA-related inflammation and cartilage catabolism. The strongest mechanistic evidence concerns IL-1β and TNF-α. However, direct cytokine targeting is not an established disease-modifying strategy in OA. Other cytokines appear to have context-dependent effects. These may vary by disease stage, joint site, and experimental model.

### 3.4. Matrix-Degrading Enzymes in Osteoarthritis: MMPs and ADAMTS

Cartilage ECM is mainly made up of two key components: type II collagen, which forms the fibrillar network, and aggrecan, a large proteoglycan that fills this network [91]. ECM degradation in articular cartilage is a key pathological process in OA, and proteolytic enzymes from the MMP and ADAMTS families play a central role in this mechanism. An imbalance between their activity and the activity of endogenous protective factors results in the progressive loss of aggrecan and type II collagen [92].

The activity of MMPs and ADAMTS is tightly regulated by pro-inflammatory cytokines and biomechanical stimuli, which promote the transition of chondrocytes toward an enhanced catabolic state, characterized by increased production of matrix-degrading proteases [25,93].

#### 3.4.1. Matrix Metalloproteinases

Increased expression of MMP-1, MMP-2 and MMP-9 has been reported in OA [94]. Synovial fluid levels of MMP-1, MMP-3, and MMP-9 showed a significant positive association with radiographic severity of knee OA assessed by the Kellgren–Lawrence grading scale (*p* < 0.001) [95].

In contrast, Rübenhagen et al. [96], reported that synovial fluid MMP-1 levels negatively correlated with OA grade. In that study, MMP-1 levels decreased with increasing disease grade. Synovial fluid concentrations of MMP-13 are elevated in OA and may serve as a biomarker of disease progression and a promising therapeutic target [97,98]. MMP-13 is the principal collagenase responsible for type II collagen cleavage and plays a central role in cartilage degradation [98,99].

MMP-1 degrades fibrillar collagens, including type I, II and III. Its overexpression in chondrocytes enhances collagen breakdown within the ECM, contributing to structural cartilage damage characteristic of OA [100,101].

Proteoglycan degradation, however, is driven primarily by aggrecanases (ADAMTS-4/5) and, to a lesser extent, by selected MMPs such as MMP-3, which also activates other MMPs and promotes broader ECM turnover [92].

In addition, the gelatinases MMP-2 and MMP-9 contribute to extracellular matrix remodeling in OA, consistent with their increased expression and activity in osteoarthritic joint tissues [94].

#### 3.4.2. A Disintegrin and Metalloproteinase with Thrombospondin Motifs

Two aggrecanases, ADAMTS-4 and ADAMTS-5, have been identified in cartilage. Among them, ADAMTS-5 is considered the predominant enzyme mediating aggrecan and other proteoglycan degradation, and its dysregulated activity has been closely linked to OA pathogenesis. Consequently, ADAMTS-5 is regarded as a key therapeutic target in the development of disease-modifying treatments for OA [102].

Majumdar et al. demonstrated that combined deletion of ADAMTS-4 and ADAMTS-5 markedly reduced proteoglycan loss in the culture of cartilage explants. In vivo, this dual deletion significantly reduced the severity of surgically induced OA in mice. Notably, the level of protection observed in double-knockout mice was comparable to that seen in mice lacking only ADAMTS-5 [103].

Cartilage degradation mediated by ADAMTS-5 releases aggrecan fragments. These fragments activate Toll-like receptor 2 (TLR2) in synovial tissue. TLR2 activation then induces inflammatory responses and pain. Together, these mechanisms establish a link between ADAMTS-5 activity and the progression of symptoms in OA [104].

Clinical trials of ADAMTS-5 inhibition have provided valuable data on safety and target engagement. Structural outcomes have also been assessed. However, a clear disease-modifying benefit in OA has not yet been established [105,106].

#### 3.4.3. Regulation of Matrix-Degrading Enzymes

**Figure 3:**
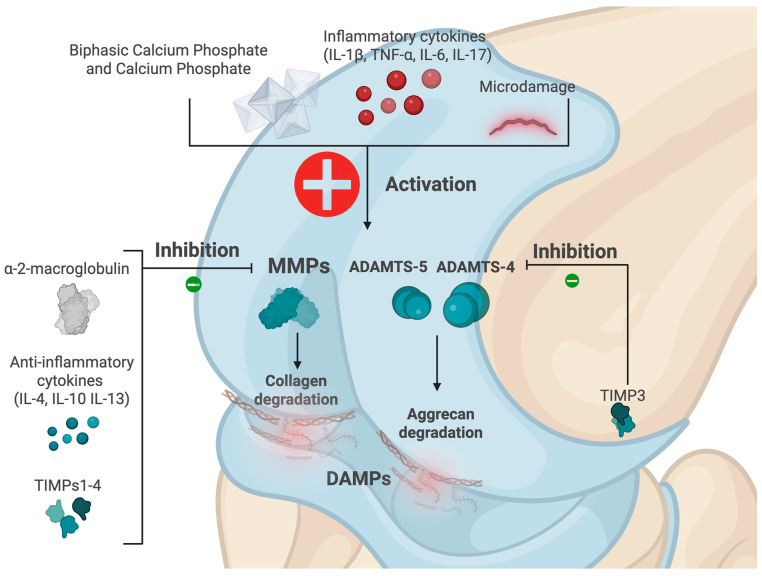
Imbalance between matrix-degrading enzymes and their inhibitors in OA. Mechanical microdamage, calcium phosphate crystals, and pro-inflammatory cytokines, including interleukin-1β (IL-1β), tumor necrosis factor alpha (TNF-α), interleukin-6 (IL-6), and interleukin-17 (IL-17), promote the activation of matrix metalloproteinases (MMPs) and A Disintegrin and Metalloproteinase with Thrombospondin Motifs (ADAMTS). MMPs mediate collagen degradation, whereas ADAMTS enzymes drive aggrecan breakdown, leading to extracellular matrix (ECM) degradation. Under physiological conditions, these enzymes are tightly regulated by tissue inhibitors of metalloproteinases (TIMPs), particularly TIMP-3, and by α2-macroglobulin, which inhibit their activity and promote their clearance. In osteoarthritis, this balance is disrupted due to increased enzyme expression and insufficient inhibitory capacity, resulting in sustained matrix degradation. Degradation products further act as damage-associated molecular patterns (DAMPs), amplifying inflammatory signaling. Created in BioRender. Bugajewski, M. (2026) https://BioRender.com/cevveuk.

The activity of matrix-degrading enzymes is regulated by a number of endogenous mechanisms that help to maintain extracellular matrix homeostasis (Figure 3). The most important of these are the tissue inhibitors of metalloproteinases (TIMPs), which regulate the function of MMPs under physiological conditions.

TIMP-1, TIMP-2, TIMP-3 and TIMP-4 bind to MMPs at a ratio of 1:1 and block their catalytic activity. TIMP-3 plays a critical role in preserving cartilage integrity. A loss of TIMP-3 accelerates collagen degradation; however, although its levels rise in OA, this increase is insufficient to counterbalance the markedly elevated MMP-13.

Another level of regulation is provided by α2-macroglobulin, which binds to active MMPs and promotes their clearance.

Aggrecanase activity is primarily controlled locally by tissue inhibitors such as TIMP-3, which directly inhibit ADAMTS-4 and ADAMTS-5. However, in OA, the balance shifts toward excessive catabolism due to the increased de novo synthesis of ADAMTS-4, which overcomes the inhibitory capacity of TIMP-3, thereby contributing to sustained aggrecan loss and cartilage destruction [98,102].

MMPs and ADAMTS enzymes are central mediators of extracellular matrix degradation in OA. However, clinical targeting of these enzymes remains difficult. Broad inhibition has not produced reliable benefit. They should therefore be interpreted as pathogenic mediators and potential biomarkers, rather than established stand-alone therapeutic targets.

### 3.5. Role of Inorganic Pyrophosphate–Inorganic Phosphate Imbalance and Basic Calcium Phosphate Crystals in Osteoarthritis

Inorganic pyrophosphate (PPi) usually inhibits the formation of hydroxyapatite, and even slight reductions in PPi can upset the equilibrium of Pi:PPi, thereby promoting the deposition of basic calcium phosphate (BCP) crystals in osteoarthritic cartilage [107].

PPi supplementation reduces ECM degradation markers and represses *Wnt5a* expression, ameliorating IL-1β-induced cartilage damage in vivo, although it does not affect synovial inflammation [108].

**Figure 4:**
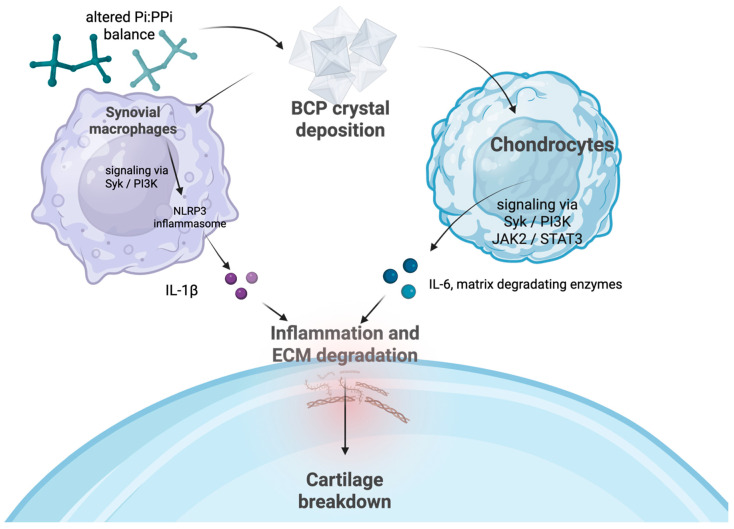
BCP crystal-driven inflammatory signaling and cartilage degradation in OA. Imbalance in PPi and Pi promotes the deposition of BCP crystals in osteoarthritic cartilage. BCP crystals activate synovial macrophages via Syk- and PI3K-dependent signaling, leading to NLRP3 inflammasome activation and IL-1β secretion. In parallel, BCP crystals stimulate chondrocytes through Syk-, PI3K-, JAK2-, and STAT3-dependent pathways, promoting IL-6 production and pro-catabolic responses. These processes converge on increased inflammatory signaling and ECM degradation, ultimately resulting in cartilage breakdown. Created in BioRender. Bugajewski, M. (2026) https://BioRender.com/b56jhbs.

BCP crystals exacerbate OA pathology by activating synovial macrophages to produce inflammatory cytokines and matrix-degrading enzymes via spleen tyrosine kinase (Syk)- and PI3K-dependent signaling [109] and inducing IL-1β secretion via NOD-like receptor protein 3 (NLRP3) inflammasome activation [110] (Figure 4).

In chondrocytes, BCP crystals stimulate IL-6 production via Syk-, PI3K-, JAK2- and STAT3-signaling pathways [111] and induce hypertrophic and Wnt/β-catenin-mediated phenotypic changes, partly by binding to Wnt3a [112] (Figure 4).

Calcium–phosphate complexes, which are elevated in early OA, further increase MMP-3 and MMP-13 through the NF-κB, p38, ERK1/2 and STAT3 pathways [113]. Taken together, these findings highlight the central role of Syk and PI3K activation in BCP-driven OA pathology and identify these kinases as potential therapeutic targets [114].

PPi–Pi imbalance and BCP crystal deposition link mineral metabolism with inflammation and cartilage degradation in OA. Experimental studies support the role of BCP-induced signaling in synovial macrophages and chondrocytes. However, their clinical relevance as therapeutic targets remains less established than their mechanistic role in OA progression.

### 3.6. Obesity, Adipokines, and Metabolic Inflammation in Osteoarthritis

Obesity is a key modifiable risk factor for osteoarthritis (OA). It contributes to OA development and progression through both mechanical and metabolic mechanisms [11,115,116]. Increased body weight raises mechanical loading in weight-bearing joints, particularly the knee and hip. However, obesity is also associated with OA in non-weight-bearing joints. This suggests that systemic metabolic inflammation also contributes to disease pathogenesis [116,117].

In obesity-associated OA, adipose tissue acts as an active endocrine and immune organ. Hypertrophic adipocytes and infiltrating immune cells promote chronic low-grade inflammation. Macrophages are particularly important in this process. This inflammatory response is partly mediated by pathogen-associated molecular patterns (PAMPs) and damage-associated molecular patterns (DAMPs). These molecules activate pattern-recognition receptors, including Toll-like receptors [117]. These pathways can increase the production of inflammatory mediators such as TNF-α, IL-6, and IL-1β. As a result, obesity may shift both the systemic environment and the local joint microenvironment toward a pro-inflammatory state.

Adipokines are important mediators linking obesity with OA pathogenesis. Leptin, resistin, chemerin, and adiponectin are among the most frequently studied adipokines in OA. Pro-inflammatory adipokines can increase cytokine production, synovial inflammation, and matrix-degrading enzyme activity. Leptin generally promotes catabolic and inflammatory responses. In contrast, the role of adiponectin is more complex and context-dependent [118]. Downstream mediators, including IL-6 and TNF-α, may further promote cartilage degradation and subchondral bone remodeling [118].

Adipokines and adipose tissue dysfunction may also contribute to OA-related pain. Adipose tissue dysfunction can influence pain through local and systemic inflammation, immune dysregulation, and adipokine production. These effects extend beyond the mechanical influence of body mass index (BMI). They may involve nociceptive pain, peripheral sensitization, and central sensitization [119].

Obesity-associated OA is supported by strong epidemiological and clinical evidence. Obesity contributes through both mechanical and metabolic mechanisms. Adipose tissue dysfunction may link obesity with inflammation, pain, and cartilage catabolism. However, the effects of individual adipokines remain partly context dependent.

### 3.7. Translational Implications of Molecular Mechanisms

The mechanisms discussed above can be linked to different clinical patterns of OA, candidate biomarkers, and potential therapeutic strategies (Table 1). This translational framework may help connect molecular pathways with patient stratification and future disease-modifying approaches. These categories are not fully separate, because several mechanisms may coexist in the same patient.

## 4. Therapeutic Perspectives and Future Directions in Osteoarthritis

Despite substantial advances in understanding the molecular mechanisms of OA, no disease-modifying osteoarthritis drugs have been established. Current treatment remains largely symptomatic. It is based mainly on non-steroidal anti-inflammatory drugs (NSAIDs) and, in advanced stages, joint replacement [120]. This gap between mechanistic insight and clinical efficacy reflects the complex nature of OA. Inflammation, matrix degradation, subchondral bone remodeling, crystal-induced signaling, mechanical loading, and metabolic dysfunction act as interconnected processes. They should not be viewed as isolated therapeutic targets. The main therapeutic targets discussed in this section, together with their mechanisms, experimental evidence, and translational limitations, are summarized in Table 2.

Several therapeutic strategies have focused on downstream inflammatory and catabolic pathways. These mechanisms remain important for understanding OA pathogenesis. However, their direct clinical targeting has produced limited benefits. Modulation of cytokine signaling, including inhibition of IL-8-mediated neutrophil recruitment and the anti-inflammatory effects of IL-37, has shown chondroprotective effects in preclinical models [121,122]. These findings have not yet translated into established clinical therapies. This may result from redundancy within inflammatory networks. Blocking a single mediator may be insufficient to suppress the overall disease process.

A similar problem applies to extracellular matrix degradation. Broad inhibition of matrix metalloproteinases has proven ineffective. More recent approaches therefore aim to rebalance protease activity rather than completely block it. Enhancing TIMP-3 activity, either through overexpression or pharmacological approaches, reduces cartilage degradation and protease activity in experimental models [123]. Similarly, α2-macroglobulin decreases MMP-13 activity and slows OA progression in preclinical models. However, its clinical benefit remains uncertain [124].

Targeting aggrecanases has also shown a clear gap between preclinical and clinical results. Inhibition of ADAMTS-4 and ADAMTS-5 reduces cartilage degradation in experimental models [102,103]. However, clinical trials have failed to show meaningful structural or symptomatic improvement [105,106]. These findings illustrate the limitations of single-target strategies in a multifactorial disease.

Subchondral bone remodeling is another mechanistically supported but clinically unresolved target. Increased RANKL activity promotes osteoclastogenesis and contributes to abnormal bone turnover in OA [40]. Although modulation of the RANK/RANKL/OPG axis has shown promise in preclinical studies, effective clinical translation has not yet been achieved. Therefore, subchondral bone remodeling remains important for understanding OA progression, but it should not be presented as a clinically validated therapeutic target.

Targeting obesity is one of the most promising approaches. It addresses both mechanical and metabolic contributors to OA. Weight loss reduces joint loading and may also modulate adipokine-related inflammation [11]. GLP-1 receptor agonists have shown greater reductions in body weight and OA-related pain than placebo in patients with knee OA [125]. Retatrutide, a triple incretin agonist targeting GLP-1, GIP, and glucagon receptors, is also being evaluated in ongoing trials in patients with knee OA [126].

Biomechanical factors remain important in OA management. Mechanical loading can influence cartilage metabolism and the expression of matrix-degrading enzymes. Experimental data suggest that physiological loading may suppress MMP expression through CBP/p300-interacting transactivator with ED-rich tail 2 [127]. However, not all physical interventions show clinical benefit. In a randomized controlled trial, low-dose radiation therapy did not improve pain, function, or inflammatory outcomes compared with placebo in patients with OA.

Crystal-induced inflammation is also relevant to OA pathogenesis. Basic calcium phosphate crystals activate Syk- and PI3K-dependent signaling in synovial macrophages, leading to increased production of inflammatory cytokines and matrix-degrading enzymes [109]. In chondrocytes, BCP crystals also stimulate IL-6 production through Syk-, PI3K-, JAK2-, and STAT3-dependent pathways [111]. These findings identify Syk and PI3K as potential therapeutic targets in BCP-driven OA pathology [114].

Biomechanical factors remain relevant in OA management. Mechanical loading influences cartilage metabolism, and experimental studies suggest that physiological loading can regulate MMP expression through CBP/p300-interacting transactivator with ED-rich tail 2 [128,129]. However, clinical translation of local physical interventions remains limited. In a randomized controlled trial, low-dose radiation therapy did not improve symptoms or inflammatory outcomes compared with placebo in patients with OA [130].

Overall, the limited clinical efficacy of previous disease-modifying strategies reflects the complexity of OA pathogenesis. Inflammatory pathways, matrix-degrading enzymes, and subchondral bone remodeling remain biologically relevant, but isolated targeting of these mechanisms has not been sufficient. Future approaches should focus on patient stratification and upstream drivers such as obesity, metabolic inflammation, and chondrocyte hypertrophic differentiation.

**Table 2:** Therapeutic targets in osteoarthritis (OA): mechanisms, experimental evidence, and translational limitations.

Therapeutic Target	Target Class/Pathway	Mechanism of Action	Evidence Level	Key Findings	Limitations	Citations
IL-8 (CXCL8)	Pro-inflammatory chemokine via CXCR1/CXCR2 receptors. Chemotaxis and inflammatory signaling axis.	Reduces CXCR1/CXCR2-mediated chemotaxis and inflammatory signaling.	Preclinical in vivo evidence; rabbit anterior cruciate ligament transection (ACLT) model	Intra articular anti IL-8 antibody reduced synovitis, joint effusion, bone marrow edema, and cartilage damage in experimental OA	Not established in OA treatment. Translation is limited by single species data, surgical OA model, and possible redundancy within chemokine signaling	[131,132]
IL-37	Anti-inflammatory cytokine via IL-18Rα and IL-1R8. Inhibition of NF-κB and MAPK pathways.	Suppresses inflammatory signaling through IL-18Rα/IL-1R8 and inhibition of NF-κB and MAPK pathways	Preclinical in vivo mouse models and in vitro human OA synovial fibroblasts	Reduced cartilage damage, osteophyte size, and joint capsule thickening in experimental OA models	Preclinical strategy. Mice lack endogenous IL-37, and adenoviral gene delivery raises durability and immunogenicity concerns.	[122,133,134]
TIMP-3 and aggrecanase selective TIMP-3 variant	Tissue inhibitors of metalloproteinases (TIMP family). Inhibition of ADAMTS aggrecanases (ADAMTS-4/5) and MMPs (for WT TIMP-3) Regulation of aggrecan and ECM breakdown.	Restores protease inhibitor balance and inhibits ADAMTS-mediated aggrecan degradation	Preclinical in vivo mouse model of surgically induced OA	TIMP-3 overexpression and aggrecanase selective TIMP-3 variants reduced cartilage degradation and OARSI scores	Transgenic overexpression is not directly translatable. Broad TIMP-3 activity may affect bone remodeling through MMP inhibition	[135]
α2-macroglobulin	Extracellular macromolecule, broad-spectrum protease inhibitor. Inhibition of active proteases (including MMPs) and inflammatory mediators (including IL-1β).	Broad protease inhibition, including MMP inhibition, and reduction in inflammatory mediators	Preclinical in vivo pig OA model and human OA-related experimental data	Early intra articular α2M injections reduced cartilage lesion area, synovitis, inflammation, and MMP-related activity	Clinical benefit remains uncertain. Available animal data are short term, and repeated injections may have tolerability limitations	[124,136]
Syk inhibition	Cytosolic tyrosine kinase; IgE/FcεRI signaling axis (mast cell activation) driving inflammatory and catabolic mediators.	Blocks Syk dependent inflammatory and catabolic signaling	Preclinical in vivo mouse model of surgically induced OA	Oral Syk inhibition reduced cartilage damage, osteophyte formation, synovitis, and expression of inflammatory and catabolic mediators	No established clinical OA data. Systemic Syk inhibition may raise safety and translation concerns	[137]
BCP crystal-induced signaling	Crystal-induced signaling axis Syk—PI3K—MAPK initiating inflammatory response.	Inhibition of Syk, PI3K, and MAPK pathways activated by BCP crystals	Mainly in vitro evidence using human primary macrophages, dendritic cells, and OA synovial fluid	BCP crystals activated Syk/PI3K signaling and increased inflammatory and catabolic mediators; pathway inhibition reduced these responses	Mechanistically relevant, but clinical therapeutic relevance remains limited. No in vivo OA efficacy data in the cited study	[114]
RANKL/OPG axis	OPG–RANK–RANKL (TNF-superfamily) osteoclastogenesis axis. RANKL by binding RANK promotes bone resorption; OPG binds RANKL and inhibits this signal.	Modulates osteoclastogenesis and subchondral bone remodeling through the OPG/RANK/RANKL pathway	Mechanistic human tissue studies and in vitro functional assays	Altered OPG/RANKL balance was associated with subchondral bone changes and osteoclast activity in OA-related models	Biologically relevant, but no OA-specific clinical treatment has been established. Donor heterogeneity and indirect functional models limit translation	[40]
ALK4/5/7 inhibition and TGF-β/SMAD modulation	TGF-β via ALK with divergent SMAD signaling. SMAD2/3 (via ALK5) versus SMAD1/5/9 (via ALK1). Crucial molecular mechanism regulating cell proliferation, differentiation, and apoptosis.	Reduces hypertrophic chondrocyte signaling while preserving more homeostatic SMAD2/3 activity	In vitro evidence using primary human OA chondrocytes	Low-dose ALK4/5/7 inhibition reduced RUNX2 and hypertrophic signaling while maintaining SMAD2/3 pathway activity	Early mechanistic strategy. Data are limited to in vitro models, and ALK4/5/7 inhibition is not pathway specific	[127]
ADAMTS-5 (aggrecanase-2)	ECM-degrading proteases—aggrecanases (ADAMTS family metalloproteases) Catabolic pathway—indicates cleavage of aggrecan, loss of proteoglycans and cartilage degeneration.	Reduces aggrecanase-mediated aggrecan cleavage and cartilage matrix degradation	Preclinical in vivo mouse model of surgically induced OA	Anti ADAMTS-5 antibody attenuated cartilage degradation, osteophyte formation, and transient mechanical allodynia	Clinical translation remains uncertain. Effects may be stage dependent, and no clear effect on subchondral bone sclerosis was reported	[138]

## 5. Conclusions

Osteoarthritis is a complex, multi-compartment disease involving cartilage, subchondral bone, and synovium. Its progression is driven by interconnected mechanisms, including chronic low-grade inflammation, extracellular matrix degradation, dysregulated bone remodeling, and disturbances in phosphate metabolism. These processes interact and sustain ongoing joint damage.

Cytokine signaling promotes catabolic activity in chondrocytes and increases osteoclastogenesis. At the same time, Pi/PPi imbalance and BCP crystal deposition enhance inflammatory responses. Together, these mechanisms lead to progressive structural deterioration of the joint.

Current treatments remain largely symptomatic and do not target the underlying drivers of disease. Progress in OA will depend on a better understanding of its molecular basis. This is necessary to identify effective therapeutic targets and develop truly disease-modifying strategies.

Further research is needed to better define these pathways and translate mechanistic insights into effective clinical therapies.

## Figures and Tables

**Table 1:** Molecular mechanisms, potential osteoarthritis (OA) phenotypes, candidate biomarkers, and therapeutic implications.

Mechanism	Potential OA Phenotype/Dominant Feature	Candidate Biomarkers or Measurable Features	Clinical Relevance in OA	Therapeutic Implications
Low-grade inflammation and cytokine signaling	Synovitis/inflammation dominant phenotype	IL-1β, TNF-α, IL-6, IL-8, synovitis on imaging	Reflects local inflammatory activity and may contribute to pain and structural progression in selected patients	Anti-inflammatory strategies and cytokine pathway modulation; direct cytokine targeting is not an established disease-modifying strategy in OA
ECM degradation mediated by MMPs and ADAMTS	Cartilage catabolic phenotype	MMP-3, MMP-13, ADAMTS-4/5 activity, aggrecan fragments, collagen degradation products	Indicates active cartilage matrix breakdown and progressive structural damage	Protease activity modulation, TIMP-related approaches, and cartilage protective strategies; broad enzyme inhibition has shown limited clinical benefit
Subchondral bone remodeling and RANK/RANKL/OPG imbalance	Subchondral bone remodeling dominant phenotype	RANKL, OPG, OPG/RANKL ratio, bone turnover markers, subchondral sclerosis, bone marrow lesions	Reflects altered bone turnover and the contribution of subchondral bone to OA progression	Bone-targeted strategies and modulation of osteoclast activity; therapeutic relevance remains mainly mechanistic or preclinical
PPi–Pi imbalance and BCP crystal deposition	Crystal/mineralization associated phenotype	PPi/Pi imbalance, BCP crystals, calcium phosphate deposition, NLRP3-related inflammatory signaling	Links mineral dysregulation with crystal-induced inflammation and cartilage degradation	Targeting mineral metabolism and crystal-induced inflammatory signaling; clinical relevance as therapeutic targets remains less established than mechanistic relevance
Obesity, adipokines, and metabolic inflammation	Metabolic/obesity-associated phenotype	BMI, metabolic syndrome markers, leptin, adiponectin, resistin, chemerin, IL-6, TNF-α	Links mechanical overload with systemic low-grade inflammation and OA-related pain	Weight loss, metabolic interventions, GLP-1 receptor agonists, and adipokine-related strategies
Chondrocyte activation, hypertrophy, and metabolic dysfunction	Chondrocyte dysfunction/hypertrophic phenotype	RUNX2, collagen X, MMP-13, altered TGF-β/SMAD signaling	Reflects loss of chondrocyte homeostasis and a shift toward cartilage catabolism	Modulation of chondrocyte phenotype, TGF-β pathway modulation, and anti-hypertrophic strategies; currently at an early translational stage

## Data Availability

No new data were created or analyzed in this study. Data sharing is not applicable to this article.

## Abbreviations

ACLT	anterior cruciate ligament transection
ADAMTS	A Disintegrin and Metalloproteinase with Thrombospondin Motifs
AKT	protein kinase B
ALK	activin receptor-like kinase
ATP	adenosine triphosphate
BCP	basic calcium phosphate
BMI	body mass index
BMPs	bone morphogenetic proteins
BV/TV	bone volume/tissue volume
CI	confidence interval
COX-2	cyclooxygenase-2
CXCL8	C-X-C motif chemokine ligand 8
CXCR1/2	C-X-C motif chemokine receptors 1 and 2
DAMPs	damage-associated molecular patterns
ECM	extracellular matrix
ERK1/2	extracellular signal-regulated kinases 1 and 2
GIP	glucose-dependent insulinotropic polypeptide
GLP-1	glucagon-like peptide-1
GM-CSF	granulocyte-macrophage colony-stimulating factor
HIF-2α	hypoxia-inducible factor 2 alpha
IL	interleukin
IL-1RA	interleukin-1 receptor antagonist
IL-4Rα	interleukin-4 receptor alpha
IL-18Rα	interleukin-18 receptor alpha
IL-18Rβ	interleukin-18 receptor beta
iNOS	inducible nitric oxide synthase
JAK	Janus kinase
JAK1	Janus kinase 1
JAK2	Janus kinase 2
LIF	leukemia inhibitory factor
MAPK	mitogen-activated protein kinase
MEK	mitogen-activated protein kinase kinase
MMPs	matrix metalloproteinases
mRNA	messenger RNA
MTF-1	metal regulatory transcription factor 1
NF-κB	nuclear factor kappa-light-chain-enhancer of activated B cells
NLRP3	NOD-like receptor protein 3
NSAIDs	non-steroidal anti-inflammatory drugs
OA	osteoarthritis
OARSI	Osteoarthritis Research Society International
OPG	osteoprotegerin
PAMPs	pathogen-associated molecular patterns
PGE2	prostaglandin E2
Pi	inorganic phosphate
PI3K	phosphoinositide 3-kinase
PLA2	phospholipase A2
PPi	inorganic pyrophosphate
RANK	receptor activator of nuclear factor kappa-B
RANKL	receptor activator of nuclear factor kappa-B ligand
ROS	reactive oxygen species
RT-qPCR	real-time quantitative polymerase chain reaction
RUNX2	runt-related transcription factor 2
SMAD	suppressor of mothers against decapentaplegic
SPECT/CT	single-photon emission computed tomography/computed tomography
STAT	signal transducer and activator of transcription
Syk	spleen tyrosine kinase
TGF-β	transforming growth factor beta
TIMPs	tissue inhibitors of metalloproteinases
TLRs	Toll-like receptors
TLR2	Toll-like receptor 2
TNF-α	tumor necrosis factor alpha
TYK2	tyrosine kinase 2
WT	wild type
α2M	alpha-2-macroglobulin

## References

[1] Hunter D.J., Bierma-Zeinstra S. (2019). Osteoarthritis. Lancet.

[2] Long H., Liu Q., Yin H., Wang K., Diao N., Zhang Y., Lin J., Guo A. (2022). Prevalence Trends of Site-Specific Osteoarthritis From 1990 to 2019: Findings From the Global Burden of Disease Study 2019. Arthritis Rheumatol..

[3] Fan Z., Yan L., Liu H., Li X., Fan K., Liu Q., Li J.J., Wang B. (2023). The prevalence of hip osteoarthritis: A systematic review and meta-analysis. Arthritis Res. Ther..

[4] Hall M., van der Esch M., Hinman R.S., Peat G., de Zwart A., Quicke J.G., Runhaar J., Knoop J., van der Leeden M., de Rooij M. (2022). How does hip osteoarthritis differ from knee osteoarthritis?. Osteoarthr. Cartil..

[5] Hannani M.T., Thudium C.S., Karsdal M.A., Ladel C., Mobasheri A., Uebelhoer M., Larkin J., Bacardit J., Struglics A., Bay-Jensen A.C. (2024). From biochemical markers to molecular endotypes of osteoarthritis: A review on validated biomarkers. Expert Rev. Mol. Diagn..

[6] Bryliński Ł., Brylińska K., Woliński F., Sado J., Smyk M., Komar O., Karpiński R., Prządka M., Baj J. (2025). Trace Elements-Role in Joint Function and Impact on Joint Diseases. Int. J. Mol. Sci..

[7] Karpiński R., Prus A., Baj J., Radej S., Prządka M., Krakowski P., Jonak K. (2025). Articular Cartilage: Structure, Biomechanics, and the Potential of Conventional and Advanced Diagnostics. Appl. Sci..

[8] Lespasio M.J., Sultan A.A., Piuzzi N.S., Khlopas A., Husni M.E., Muschler G.F., Mont M.A. (2018). Hip Osteoarthritis: A Primer. Perm. J..

[9] Tang S., Zhang C., Oo W.M., Fu K., Risberg M.A., Bierma-Zeinstra S.M., Neogi T., Atukorala I., Malfait A.M., Ding C. (2025). Osteoarthritis. Nat. Rev. Dis. Primers.

[10] Di Cicco G., Marzano E., Mastrostefano A., Pitocco D., Castilho R.S., Zambelli R., Mascio A., Greco T., Cinelli V., Comisi C. (2024). The Pathogenetic Role of RANK/RANKL/OPG Signaling in Osteoarthritis and Related Targeted Therapies. Biomedicines.

[11] Kloppenburg M., Namane M., Cicuttini F. (2025). Osteoarthritis. Lancet.

[12] Runhaar J., Özbulut Ö., Kloppenburg M., Boers M., Bijlsma J.W.J., Bierma-Zeinstra S.M.A. (2021). CREDO expert group. Diagnostic criteria for early hip osteoarthritis: First steps, based on the CHECK study. Rheumatology.

[13] Abramoff B., Caldera F.E. (2020). Osteoarthritis: Pathology, Diagnosis, and Treatment Options. Med. Clin. N. Am..

[14] Chow Y.Y., Chin K.Y. (2020). The Role of Inflammation in the Pathogenesis of Osteoarthritis. Mediat. Inflamm..

[15] Sen R., Hurley J.A. (2025). Osteoarthritis. StatPearls.

[16] Bobinac D., Spanjol J., Zoricic S., Maric I. (2003). Changes in articular cartilage and subchondral bone histomorphometry in osteoarthritic knee joints in humans. Bone.

[17] Yao Q., Wu X., Tao C., Gong W., Chen M., Qu M., Zhong Y., He T., Chen S., Xiao G. (2023). Osteoarthritis: Pathogenic signaling pathways and therapeutic targets. Signal Transduct. Target. Ther..

[18] Yunus M.H.M., Nordin A., Kamal H. (2020). Pathophysiological Perspective of Osteoarthritis. Medicina.

[19] Choi M.C., Jo J., Park J., Kang H.K., Park Y. (2019). NF-kappaB Signaling Pathways in Osteoarthritic Cartilage Destruction. Cells.

[20] Saito T., Fukai A., Mabuchi A., Ikeda T., Yano F., Ohba S., Nishida N., Akune T., Yoshimura N., Nakagawa T. (2010). Transcriptional regulation of endochondral ossification by HIF-2alpha during skeletal growth and osteoarthritis development. Nat. Med..

[21] Kim J.H., Jeon J., Shin M., Won Y., Lee M., Kwak J.S., Lee G., Rhee J., Ryu J.H., Chun C.H. (2014). Regulation of the catabolic cascade in osteoarthritis by the zinc-ZIP8-MTF1 axis. Cell.

[22] Mobasheri A., Batt M. (2016). An update on the pathophysiology of osteoarthritis. Ann. Phys. Rehabil. Med..

[23] Molnar V., Matišić V., Kodvanj I., Bjelica R., Jeleč Ž., Hudetz D., Rod E., Čukelj F., Vrdoljak T., Vidović D. (2021). Cytokines and Chemokines Involved in Osteoarthritis Pathogenesis. Int. J. Mol. Sci..

[24] Kunisch E., Kinne R.W., Alsalameh R.J., Alsalameh S. (2016). Pro-inflammatory IL-1beta and/or TNF-alpha up-regulate matrix metalloproteases-1 and -3 mRNA in chondrocyte subpopulations potentially pathogenic in osteoarthritis: In situ hybridization studies on a single cell level. Int. J. Rheum. Dis..

[25] Kapoor M., Martel-Pelletier J., Lajeunesse D., Pelletier J.P., Fahmi H. (2011). Role of proinflammatory cytokines in the pathophysiology of osteoarthritis. Nat. Rev. Rheumatol..

[26] Xia B., Chen D., Zhang J., Hu S., Jin H., Tong P. (2014). Osteoarthritis pathogenesis: A review of molecular mechanisms. Calcif. Tissue Int..

[27] Donell S. (2019). Subchondral bone remodelling in osteoarthritis. EFORT Open Rev..

[28] Li G., Yin J., Gao J., Cheng T.S., Pavlos N.J., Zhang C., Zheng M.H. (2013). Subchondral bone in osteoarthritis: Insight into risk factors and microstructural changes. Arthritis Res. Ther..

[29] Delsmann J., Eissele J., Simon A., Alimy A.R., von Kroge S., Mushumba H., Püschel K., Busse B., Ries C., Amling M. (2024). Alterations in compositional and cellular properties of the subchondral bone are linked to cartilage degeneration in hip osteoarthritis. Osteoarthr. Cartil..

[30] Sanchez-Lopez E., Coras R., Torres A., Lane N.E., Guma M. (2022). Synovial inflammation in osteoarthritis progression. Nat. Rev. Rheumatol..

[31] Xiong J., Piemontese M., Onal M., Campbell J., Goellner J.J., Dusevich V., Bonewald L., Manolagas S.C., O’Brien C.A. (2015). Osteocytes, not Osteoblasts or Lining Cells, are the Main Source of the RANKL Required for Osteoclast Formation in Remodeling Bone. PLoS ONE.

[32] Hwang H.S., Park S.J., Cheon E.J., Lee M.H., Kim H.A. (2015). Fibronectin fragment-induced expression of matrix metalloproteinases is mediated by MyD88-dependent TLR-2 signaling pathway in human chondrocytes. Arthritis Res. Ther..

[33] Goldring S.R., Scanzello C.R. (2012). Plasma proteins take their toll on the joint in osteoarthritis. Arthritis Res. Ther..

[34] Verborgt O., Gibson G.J., Schaffler M.B. (2000). Loss of osteocyte integrity in association with microdamage and bone remodeling after fatigue in vivo. J. Bone Min. Res..

[35] Kennedy O.D., Laudier D.M., Majeska R.J., Sun H.B., Schaffler M.B. (2014). Osteocyte apoptosis is required for production of osteoclastogenic signals following bone fatigue in vivo. Bone.

[36] Kennedy O.D., Herman B.C., Laudier D.M., Majeska R.J., Sun H.B., Schaffler M.B. (2012). Activation of resorption in fatigue-loaded bone involves both apoptosis and active pro-osteoclastogenic signaling by distinct osteocyte populations. Bone.

[37] Nakashima T., Hayashi M., Fukunaga T., Kurata K., Oh-Hora M., Feng J.Q., Bonewald L.F., Kodama T., Wutz A., Wagner E.F. (2011). Evidence for osteocyte regulation of bone homeostasis through RANKL expression. Nat. Med..

[38] Martinez-Calatrava M.J., Prieto-Potin I., Roman-Blas J.A., Tardio L., Largo R., Herrero-Beaumont G. (2012). RANKL synthesized by articular chondrocytes contributes to juxta-articular bone loss in chronic arthritis. Arthritis Res. Ther..

[39] Walsh N.C., Alexander K.A., Manning C.A., Karmakar S., Wang J.F., Weyand C.M., Pettit A.R., Gravallese E.M. (2013). Activated human T cells express alternative mRNA transcripts encoding a secreted form of RANKL. Genes Immun..

[40] Kwan Tat S., Pelletier J.P., Lajeunesse D., Fahmi H., Lavigne M., Martel-Pelletier J. (2008). The differential expression of osteoprotegerin (OPG) and receptor activator of nuclear factor kappaB ligand (RANKL) in human osteoarthritic subchondral bone osteoblasts is an indicator of the metabolic state of these disease cells. Clin. Exp. Rheumatol..

[41] Chou C.H., Wu C.C., Song I.W., Chuang H.P., Lu L.S., Chang J.H., Kuo S.Y., Lee C.H., Wu J.Y., Chen Y.T. (2013). Genome-wide expression profiles of subchondral bone in osteoarthritis. Arthritis Res. Ther..

[42] Findlay D., Chehade M., Tsangari H., Neale S., Hay S., Hopwood B., Pannach S., O’Loughlin P., Fazzalari N. (2008). Circulating RANKL is inversely related to RANKL mRNA levels in bone in osteoarthritic males. Arthritis Res. Ther..

[43] Goldring M.B., Otero M. (2011). Inflammation in osteoarthritis. Curr. Opin. Rheumatol..

[44] Kwan Tat S., Amiable N., Pelletier J.P., Boileau C., Lajeunesse D., Duval N., Martel-Pelletier J. (2009). Modulation of OPG, RANK and RANKL by human chondrocytes and their implication during osteoarthritis. Rheumatology.

[45] Hashizume M., Hayakawa N., Mihara M. (2008). IL-6 trans-signalling directly induces RANKL on fibroblast-like synovial cells and is involved in RANKL induction by TNF-alpha and IL-17. Rheumatology.

[46] De Roover A., Escribano-Nunez A., Monteagudo S., Lories R. (2023). Fundamentals of osteoarthritis: Inflammatory mediators in osteoarthritis. Osteoarthr. Cartil..

[47] Wojdasiewicz P., Poniatowski L.A., Szukiewicz D. (2014). The role of inflammatory and anti-inflammatory cytokines in the pathogenesis of osteoarthritis. Mediat. Inflamm..

[48] Moos V., Fickert S., Müller B., Weber U., Sieper J. (1999). Immunohistological analysis of cytokine expression in human osteoarthritic and healthy cartilage. J. Rheumatol..

[49] Thielen N.G.M., van der Kraan P.M., van Caam A.P.M. (2019). TGFbeta/BMP Signaling Pathway in Cartilage Homeostasis. Cells.

[50] Thielen N., Neefjes M., Wiegertjes R., van den Akker G., Vitters E., van Beuningen H., Blaney Davidson E., Koenders M., van Lent P., van de Loo F. (2021). Osteoarthritis-Related Inflammation Blocks TGF-beta’s Protective Effect on Chondrocyte Hypertrophy via (de)Phosphorylation of the SMAD2/3 Linker Region. Int. J. Mol. Sci..

[51] Du X., Cai L., Xie J., Zhou X. (2023). The role of TGF-beta3 in cartilage development and osteoarthritis. Bone Res..

[52] Lopez-Armada M.J., Carames B., Martin M.A., Cillero-Pastor B., Lires-Dean M., Fuentes-Boquete I., Arenas J., Blanco F.J. (2006). Mitochondrial activity is modulated by TNFalpha and IL-1beta in normal human chondrocyte cells. Osteoarthr. Cartil..

[53] Benito M.J., Veale D.J., FitzGerald O., van den Berg W.B., Bresnihan B. (2005). Synovial tissue inflammation in early and late osteoarthritis. Ann. Rheum. Dis..

[54] Sellam J., Berenbaum F. (2010). The role of synovitis in pathophysiology and clinical symptoms of osteoarthritis. Nat. Rev. Rheumatol..

[55] Ansari M.Y., Ahmad N., Haqqi T.M. (2020). Oxidative stress and inflammation in osteoarthritis pathogenesis: Role of polyphenols. Biomed. Pharmacother..

[56] Pulsatelli L., Dolzani P., Piacentini A., Silvestri T., Ruggeri R., Gualtieri G., Meliconi R., Facchini A. (1999). Chemokine production by human chondrocytes. J. Rheumatol..

[57] Lotz M., Terkeltaub R., Villiger P.M. (1992). Cartilage and joint inflammation. Regulation of IL-8 expression by human articular chondrocytes. J. Immunol..

[58] Favero M., Belluzzi E., Trisolino G., Goldring M.B., Goldring S.R., Cigolotti A., Pozzuoli A., Ruggieri P., Ramonda R., Grigolo B. (2019). Inflammatory molecules produced by meniscus and synovium in early and end-stage osteoarthritis: A coculture study. J. Cell Physiol..

[59] Abe H., Sakai T., Ando W., Takao M., Nishii T., Nakamura N., Hamasaki T., Yoshikawa H., Sugano N. (2014). Synovial joint fluid cytokine levels in hip disease. Rheumatology.

[60] Koh S.M., Chan C.K., Teo S.H., Singh S., Merican A., Ng W.M., Abbas A., Kamarul T. (2020). Elevated plasma and synovial fluid interleukin-8 and interleukin-18 may be associated with the pathogenesis of knee osteoarthritis. Knee.

[61] Moreau M., Brocheriou I., Petit L., Ninio E., Chapman M.J., Rouis M. (1999). Interleukin-8 mediates downregulation of tissue inhibitor of metalloproteinase-1 expression in cholesterol-loaded human macrophages: Relevance to stability of atherosclerotic plaque. Circulation.

[62] Shi B., Guo X., Iv A., Zhang Z., Shi X. (2022). Polymorphism of MMP-3 gene and imbalance expression of MMP-3/TIMP-1 in articular cartilage are associated with an endemic osteochondropathy, Kashin- Beck disease. BMC Musculoskelet. Disord..

[63] Snelling S.J.B., Bas S., Puskas G.J., Dakin S.G., Suva D., Finckh A., Gabay C., Hoffmeyer P., Carr A.J., Lübbeke A. (2017). Presence of IL-17 in synovial fluid identifies a potential inflammatory osteoarthritic phenotype. PLoS ONE.

[64] Yang H.Y., Liu Y.Z., Zhou X.D., Huang Y., Xu N.W. (2020). Role of IL-17 gene polymorphisms in osteoarthritis: A meta-analysis based on observational studies. World J. Clin. Cases.

[65] Sinkeviciute D., Aspberg A., He Y., Bay-Jensen A.C., Önnerfjord P. (2020). Characterization of the interleukin-17 effect on articular cartilage in a translational model: An explorative study. BMC Rheumatol..

[66] Chabaud M., Lubberts E., Joosten L., van Den Berg W., Miossec P. (2001). IL IL-17 derived from juxta-articular bone and synovium contributes to joint degradation in rheumatoid arthritis. Arthritis Res..

[67] Chyuan I.T., Chen J.Y. (2018). Role of Interleukin- (IL-) 17 in the Pathogenesis and Targeted Therapies in Spondyloarthropathies. Mediat. Inflamm..

[68] Yong K.L., Rowles P.M., Patterson K.G., Linch D.C. (1992). Granulocyte-macrophage colony-stimulating factor induces neutrophil adhesion to pulmonary vascular endothelium in vivo: Role of beta 2 integrins. Blood.

[69] Weisbart R.H., Kwan L., Golde D.W., Gasson J.C. (1987). Human Human GM-CSF primes neutrophils for enhanced oxidative metabolism in response to the major physiological chemoattractants. Blood.

[70] Lopez A.F., Williamson D.J., Gamble J.R., Begley C.G., Harlan J.M., Klebanoff S.J., Waltersdorph A., Wong G., Clark S.C., Vadas M.A. (1986). Recombinant human granulocyte-macrophage colony-stimulating factor stimulates in vitro mature human neutrophil and eosinophil function, surface receptor expression, and survival. J. Clin. Investig..

[71] Zhang H., Cai D., Bai X. (2020). Macrophages regulate the progression of osteoarthritis. Osteoarthr. Cartil..

[72] Bondeson J., Wainwright S.D., Lauder S., Amos N., Hughes C.E. (2006). The role of synovial macrophages and macrophage-produced cytokines in driving aggrecanases, matrix metalloproteinases, and other destructive and inflammatory responses in osteoarthritis. Arthritis Res. Ther..

[73] Wang T., He C. (2018). Pro-inflammatory cytokines: The link between obesity and osteoarthritis. Cytokine Growth Factor Rev..

[74] Beekhuizen M., Gierman L.M., van Spil W.E., van Osch G.J.V.M., Huizinga T.W.J., Saris D.B.F., Creemers L.B., Zuurmond A.M. (2013). An explorative study comparing levels of soluble mediators in control and osteoarthritic synovial fluid. Osteoarthr. Cartil..

[75] Goekoop R.J., Kloppenburg M., Kroon H.M., Frolich M., Huizinga T.W.J., Westendorp R.G.J., Gussekloo J. (2010). Low innate production of interleukin-1beta and interleukin-6 is associated with the absence of osteoarthritis in old age. Osteoarthr. Cartil..

[76] de Hooge A.S.K., van de Loo F.A.J., Bennink M.B., Arntz O.J., de Hooge P., van den Berg W.B. (2005). Male IL-6 gene knock out mice developed more advanced osteoarthritis upon aging. Osteoarthr. Cartil..

[77] Li L., Li Z., Li Y., Hu X., Zhang Y., Fan P. (2020). Profiling of inflammatory mediators in the synovial fluid related to pain in knee osteoarthritis. BMC Musculoskelet. Disord..

[78] Mueller T.D., Zhang J.L., Sebald W., Duschl A. (2002). Structure, binding, and antagonists in the IL-4/IL-13 receptor system. Biochim. Biophys. Acta.

[79] Silvestri T., Pulsatelli L., Dolzani P., Facchini A., Meliconi R. (2006). Elevated serum levels of soluble interleukin-4 receptor in osteoarthritis. Osteoarthr. Cartil..

[80] Doi H., Nishida K., Yorimitsu M., Komiyama T., Kadota Y., Tetsunaga T., Yoshida A., Kubota S., Takigawa M., Ozaki T. (2008). Interleukin-4 downregulates the cyclic tensile stress-induced matrix metalloproteinases-13 and cathepsin B expression by rat normal chondrocytes. Acta Med. Okayama.

[81] van Meegeren M.E., Roosendaal G., Jansen N.W., Wenting M.J., van Wesel A.C., van Roon J.A., Lafeber F.P. (2012). IL-4 alone and in combination with IL-10 protects against blood-induced cartilage damage. Osteoarthr. Cartil..

[82] Alaaeddine N., Di Battista J.A., Pelletier J.P., Kiansa K., Cloutier J.M., Martel-Pelletier J. (1999). Inhibition of tumor necrosis factor alpha-induced prostaglandin E2 production by the antiinflammatory cytokines interleukin-4, interleukin-10, and interleukin-13 in osteoarthritic synovial fibroblasts: Distinct targeting in the signaling pathways. Arthritis Rheum..

[83] Nees T.A., Rosshirt N., Zhang J.A., Reiner T., Sorbi R., Tripel E., Walker T., Schiltenwolf M., Hagmann S., Moradi B. (2019). Synovial Cytokines Significantly Correlate with Osteoarthritis-Related Knee Pain and Disability: Inflammatory Mediators of Potential Clinical Relevance. J. Clin. Med..

[84] Moue T., Tajika Y., Ishikawa S., Kanada Y., Okumo T., Asano K., Hisamitsu T. (2017). Influence of IL13 on Periostin Secretion by Synoviocytes in Osteoarthritis. In Vivo.

[85] Tajika Y., Moue T., Ishikawa S., Asano K., Okumo T., Takagi H., Hisamitsu T. (2017). Influence of Periostin on Synoviocytes in Knee Osteoarthritis. In Vivo.

[86] Nabbe K.C., van Lent P.L., Holthuysen A.E., Sloëtjes A.W., Koch A.E., Radstake T.R., van den Berg W.B. (2005). Local IL-13 gene transfer prior to immune-complex arthritis inhibits chondrocyte death and matrix-metalloproteinase-mediated cartilage matrix degradation despite enhanced joint inflammation. Arthritis Res. Ther..

[87] Zdanov A., Schalk-Hihi C., Wlodawer A. (1995). Crystal structure of interleukin-10 reveals the functional dimer with an unexpected topological similarity to interferon gamma. Structure.

[88] Lacraz S., Nicod L.P., Chicheportiche R., Welgus H.G., Dayer J.M. (1995). IL-10 inhibits metalloproteinase and stimulates TIMP-1 production in human mononuclear phagocytes. J. Clin. Investig..

[89] Behrendt P., Preusse-Prange A., Klüter T., Haake M., Rolauffs B., Grodzinsky A.J., Lippross S., Kurz B. (2016). IL-10 reduces apoptosis and extracellular matrix degradation after injurious compression of mature articular cartilage. Osteoarthr. Cartil..

[90] Jung Y.K., Kim G.W., Park H.R., Lee E.J., Choi J.Y., Beier F., Han S.W. (2013). Role of interleukin-10 in endochondral bone formation in mice: Anabolic effect via the bone morphogenetic protein/Smad pathway. Arthritis Rheum..

[91] Goldring M.B. (2000). The role of the chondrocyte in osteoarthritis. Arthritis Rheum..

[92] Troeberg L., Nagase H. (2012). Proteases involved in cartilage matrix degradation in osteoarthritis. Biochim. Biophys. Acta.

[93] Guilak F., Nims R.J., Dicks A., Wu C.L., Meulenbelt I. (2018). Osteoarthritis as a disease of the cartilage pericellular matrix. Matrix Biol..

[94] Zeng G.Q., Chen A.B., Li W., Song J.H., Gao C.Y. (2015). High MMP-1, MMP-2, and MMP-9 protein levels in osteoarthritis. Genet. Mol. Res..

[95] Kumar S., Kumar H., Mittal A., Singh P.P., Yadav V., Kumar D., Ahmad I., Mishra V. (2023). Correlation Between Synovial Fluid Levels of Matrix Metalloproteinase’s (MMP-1, MMP-3, and MMP-9) and TNF-alpha with the Severity of Osteoarthritis Knee in Rural Indian Population. Indian J. Orthop..

[96] Rubenhagen R., Schuttrumpf J.P., Sturmer K.M., Frosch K.H. (2012). Interleukin-7 levels in synovial fluid increase with age and MMP-1 levels decrease with progression of osteoarthritis. Acta Orthop..

[97] Kumar P., Kumar S., Abhilasha A., Singh A., Kumar U. (2023). The Role of Matrix Metalloproteinase 13 and Vitamin D in Osteoarthritis: A Hospital-Based Observational Study. Cureus.

[98] Hu Q., Ecker M. (2021). Overview of MMP-13 as a Promising Target for the Treatment of Osteoarthritis. Int. J. Mol. Sci..

[99] Rowan A.D., Litherland G.J., Hui W., Milner J.M. (2008). Metalloproteases as potential therapeutic targets in arthritis treatment. Expert. Opin. Ther. Targets.

[100] Kaspiris A., Khaldi L., Grivas T.B., Vasiliadis E., Kouvaras I., Dagkas S., Chronopoulos E., Papadimitriou E. (2013). Subchondral cyst development and MMP-1 expression during progression of osteoarthritis: An immunohistochemical study. Orthop. Traumatol. Surg. Res..

[101] Tetlow L.C., Adlam D.J., Woolley D.E. (2001). Matrix metalloproteinase and proinflammatory cytokine production by chondrocytes of human osteoarthritic cartilage: Associations with degenerative changes. Arthritis Rheum..

[102] Verma P., Dalal K. (2011). ADAMTS-4 and ADAMTS-5: Key enzymes in osteoarthritis. J. Cell Biochem..

[103] Majumdar M.K., Askew R., Schelling S., Stedman N., Blanchet T., Hopkins B., Morris E.A., Glasson S.S. (2007). Double-knockout of ADAMTS-4 and ADAMTS-5 in mice results in physiologically normal animals and prevents the progression of osteoarthritis. Arthritis Rheum..

[104] Sharma N., Drobinski P., Kayed A., Chen Z., Kjelgaard-Petersen C.F., Gantzel T., Karsdal M.A., Michaelis M., Ladel C., Bay-Jensen A.C. (2020). Inflammation and joint destruction may be linked to the generation of cartilage metabolites of ADAMTS-5 through activation of toll-like receptors. Osteoarthr. Cartil..

[105] Schnitzer T., Pueyo M., Deckx H., van der Aar E., Bernard K., Hatch S., van der Stoep M., Grankov S., Phung D., Imbert O. (2023). Evaluation of S201086/GLPG1972, an ADAMTS-5 inhibitor, for the treatment of knee osteoarthritis in ROCCELLA: A phase 2 randomized clinical trial. Osteoarthr. Cartil..

[106] Bihlet A.R., Balchen T., Goteti K., Sonne J., Ladel C., Karsdal M.A., Ona V., Moreau F., Waterhouse R., Bay-Jensen A.C. (2024). Safety, Tolerability, and Pharmacodynamics of the ADAMTS-5 Nanobody M6495: Two Phase 1, Single-Center, Double-Blind, Randomized, Placebo-Controlled Studies in Healthy Subjects and Patients with Osteoarthritis. ACR Open Rheumatol..

[107] Bernabei I., So A., Busso N., Nasi S. (2023). Cartilage calcification in osteoarthritis: Mechanisms and clinical relevance. Nat. Rev. Rheumatol..

[108] Velot E., Guibert M., Koufany M., Bianchi A. (2025). Intra-articular injection of inorganic pyrophosphate improves IL-1beta-induced cartilage damage in rat model of knee osteoarthritis in vivo. Osteoarthr. Cart. Open.

[109] Cunningham C.C., Mills E., Mielke L.A., O’Farrell L.K., Lavelle E., Mori A., McCarthy G.M., Mills K.H., Dunne A. (2012). Osteoarthritis-associated basic calcium phosphate crystals induce pro-inflammatory cytokines and damage-associated molecules via activation of Syk and PI3 kinase. Clin. Immunol..

[110] Pazár B., Ea H.K., Narayan S., Kolly L., Bagnoud N., Chobaz V., Roger T., Lioté F., So A., Busso N. (2011). Basic calcium phosphate crystals induce monocyte/macrophage IL-1beta secretion through the NLRP3 inflammasome in vitro. J. Immunol..

[111] Nasi S., So A., Combes C., Daudon M., Busso N. (2016). Interleukin-6 and chondrocyte mineralisation act in tandem to promote experimental osteoarthritis. Ann. Rheum. Dis..

[112] Bertrand J., Kräft T., Gronau T., Sherwood J., Rutsch F., Lioté F., Dell’Accio F., Lohmann C.H., Bollmann M., Held A. (2020). BCP crystals promote chondrocyte hypertrophic differentiation in OA cartilage by sequestering Wnt3a. Ann. Rheum. Dis..

[113] Jung Y.K., Han M.S., Park H.R., Lee E.J., Jang J.A., Kim G.W., Lee S.Y., Moon D., Han S. (2018). Calcium-phosphate complex increased during subchondral bone remodeling affects earlystage osteoarthritis. Sci. Rep..

[114] Corr E.M., Cunningham C.C., Helbert L., McCarthy G.M., Dunne A. (2017). Osteoarthritis-associated basic calcium phosphate crystals activate membrane proximal kinases in human innate immune cells. Arthritis Res. Ther..

[115] Godziuk K., Hawker G.A. (2024). Obesity and body mass index: Past and future considerations in osteoarthritis research. Osteoarthr. Cartil..

[116] Thijssen E., van Caam A., van der Kraan P.M. (2015). Obesity and osteoarthritis, more than just wear and tear: Pivotal roles for inflamed adipose tissue and dyslipidaemia in obesity-induced osteoarthritis. Rheumatology.

[117] Nedunchezhiyan U., Varughese I., Sun A.R., Wu X., Crawford R., Prasadam I. (2022). Obesity, Inflammation, and Immune System in Osteoarthritis. Front. Immunol..

[118] Xie C., Chen Q. (2019). Adipokines: New Therapeutic Target for Osteoarthritis?. Curr. Rheumatol. Rep..

[119] Binvignat M., Sellam J., Berenbaum F., Felson D.T. (2024). The role of obesity and adipose tissue dysfunction in osteoarthritis pain. Nat. Rev. Rheumatol..

[120] Misra D., Felson D.T. (2024). Evidence-Based Review of Nonsurgical Treatments for Knee and Hip Osteoarthritis. Eur. J. Rheumatol..

[121] Harada A., Sekido N., Akahoshi T., Wada T., Mukaida N., Matsushima K. (1994). Essential involvement of interleukin-8 (IL-8) in acute inflammation. J. Leukoc. Biol..

[122] van Geffen E.W., van Caam A.P.M., van Beuningen H.M., Vitters E.L., Schreurs W., van de Loo F.A., van Lent P.L., Koenders M.I., Blaney Davidson E.N., van der Kraan P.M. (2017). IL37 dampens the IL1beta-induced catabolic status of human OA chondrocytes. Rheumatology.

[123] Meszaros E., Malemud C.J. (2012). Prospects for treating osteoarthritis: Enzyme-protein interactions regulating matrix metalloproteinase activity. Ther. Adv. Chronic Dis..

[124] Wang S., Wei X., Zhou J., Zhang J., Li K., Chen Q., Terek R., Fleming B.C., Goldring M.B., Ehrlich M.G. (2014). Identification of alpha2-macroglobulin as a master inhibitor of cartilage-degrading factors that attenuates the progression of posttraumatic osteoarthritis. Arthritis Rheumatol..

[125] Bliddal H., Bays H., Czernichow S., Uddén Hemmingsson J., Hjelmesæth J., Hoffmann Morville T., Koroleva A., Skov Neergaard J., Vélez Sánchez P., Wharton S. (2024). Once-Weekly Semaglutide in Persons with Obesity and Knee Osteoarthritis. N. Engl. J. Med..

[126] Giblin K., Kaplan L.M., Somers V.K., Le Roux C.W., Hunter D.J., Wu Q., Lalonde A., Ahmad N., Bethel M.A. (2026). Retatrutide for the treatment of obesity, obstructive sleep apnea and knee osteoarthritis: Rationale and design of the TRIUMPH registrational clinical trials. Diabetes Obes. Metab..

[127] Thielen N.G.M., van Caam A.P.M., van Beuningen H.M., Vitters E.L., van den Bosch M.H.J., Koenders M.I., van de Loo F.A.J., Blaney Davidson E.N., van der Kraan P.M. (2023). Separating friend from foe: Inhibition of TGF-beta-induced detrimental SMAD1/5/9 phosphorylation while maintaining protective SMAD2/3 signaling in OA chondrocytes. Osteoarthr. Cartil..

[128] Leong D.J., Li Y.H., Gu X.I., Sun L., Zhou Z., Nasser P., Laudier D.M., Iqbal J., Majeska R.J., Schaffler M.B. (2011). Physiological loading of joints prevents cartilage degradation through CITED2. FASEB J..

[129] Kanakis I., Liu K., Poulet B., Javaheri B., van ’t Hof R.J., Pitsillides A.A., Bou-Gharios G. (2019). Targeted Inhibition of Aggrecanases Prevents Articular Cartilage Degradation and Augments Bone Mass in the STR/Ort Mouse Model of Spontaneous Osteoarthritis. Arthritis Rheumatol..

[130] Mahler E.A.M., Minten M.J.M., Leseman-Hoogenboom M.M., Poortmans P.M.P., Leer J.W.H., Boks S.S., van den Hoogen F.H.J., den Broeder A.A., van den Ende C.H.M. (2019). Effectiveness of low-dose radiation therapy on symptoms in patients with knee osteoarthritis: A randomised, double-blinded, sham-controlled trial. Ann. Rheum. Dis..

[131] Yang J., Wang X., Zhang Y., He R., Fu Z., Wang R., Ma Y., Fu D., Meng S., Cai W. (2025). Intra-Articular Injection of Interleukin-8 Neutralizing Monoclonal Antibody Effectively Attenuates Osteoarthritis Progression in Rabbits. Cartilage.

[132] Bilusic M., Heery C.R., Collins J.M., Donahue R.N., Palena C., Madan R.A., Karzai F., Marté J.L., Strauss J., Gatti-Mays M.E. (2019). Phase I trial of HuMax-IL8 (BMS-986253), an anti-IL-8 monoclonal antibody, in patients with metastatic or unresectable solid tumors. J. Immunother. Cancer.

[133] Su Z., Tao X. (2021). Current Understanding of IL-37 in Human Health and Disease. Front. Immunol..

[134] van Geffen E.W., van Beuningen H.M., Aarts J., Vitters E.L., Rijnen W.H.C., Blom A.B., van de Loo F.A.J., Blaney Davidson E.N., Koenders M.I., van Caam A.P.M. (2025). Interleukin-37 Ameliorates Articular Cartilage Damage in Two Murine Models of Osteoarthritis. Cartilage.

[135] Nakamura H., Vo P., Kanakis I., Liu K., Bou-Gharios G. (2020). Aggrecanase-selective tissue inhibitor of metalloproteinase-3 (TIMP3) protects articular cartilage in a surgical mouse model of osteoarthritis. Sci. Rep..

[136] Sun C., Chang K., Fleming B.C., Owens B.D., Beveridge J.E., Zhao Y., Peng G., Wei L. (2024). Alpha-2-Macroglobulin Attenuates Posttraumatic Osteoarthritis Cartilage Damage by Inhibiting Inflammatory Pathways With Modified Intra-articular Drilling in a Yucatan Minipig Model. Am. J. Sports Med..

[137] Wang Q., Lepus C.M., Raghu H., Reber L.L., Tsai M.M., Wong H.H., von Kaeppler E., Lingampalli N., Bloom M.S., Hu N. (2019). IgE-mediated mast cell activation promotes inflammation and cartilage destruction in osteoarthritis. Elife.

[138] Miller R.E., Tran P.B., Ishihara S., Larkin J., Malfait A.M. (2016). Therapeutic effects of an anti-ADAMTS-5 antibody on joint damage and mechanical allodynia in a murine model of osteoarthritis. Osteoarthr. Cartil..

